# Efficacy and safety of intravenous immunoglobulins for the treatment of viral encephalitis: a systematic literature review

**DOI:** 10.1007/s00415-021-10494-w

**Published:** 2021-03-06

**Authors:** Judith N. Wagner, Annette Leibetseder, Anna Troescher, Juergen Panholzer, Tim J. von Oertzen

**Affiliations:** Department of Neurology 1, Kepler University Hospital, Johannes Kepler University, Wagner-Jauregg-Weg 15, 4020 Linz, Austria

**Keywords:** Encephalitis, Viral infections, Immunoglobulins, Critical care

## Abstract

**Background:**

For most viral encephalitides, therapy is merely supportive. Intravenous immunoglobulins (IVIG) have been used as a prophylactic and therapeutic approach. We conduct a systematic review on the safety and efficacy of IVIG in viral encephalitis.

**Methods:**

We conducted a systematic review assessing PubMed, Cochrane Database, Biosis Previews and the ClinicalTrials.gov website to identify all reports on patients with viral encephalitis treated with IVIG as of May 31, 2019. The main outcomes assessed were therapeutic efficacy and safety. For an increased homogeneity of the population, atypical viral infections were excluded, as were reports on prophylactic IVIG use, intrathecal application of immunoglobulins, or use of antibody-enriched IVIG-preparations. Data were extracted from published studies. Descriptive statistics were used.

**Results:**

We included a total of 44 studies (39 case reports). The case reports cover a total of 53 patients. Our search retrieved two prospective and three retrospective studies. These show heterogeneous results as to the efficacy of IVIG therapy. Only one study reports a significant association between IVIG-use and death (odds ratio 0.032; 95% confidence interval 0.0033–0.3024; *p* = 0.0027). None of the studies report significant differences in the number of serious adverse events.

**Conclusion:**

Data on the efficacy of IVIG-therapy is heterogeneous. While it seems generally safe, evident superiority compared to supportive treatment has not been demonstrated so far. Future trials should also investigate the optimal dosing and timing of IVIG and their benefit in the immunosuppressed.

## Introduction

Encephalitis is an acute neurological syndrome characterized by altered mental status in combination with two or more secondary diagnostic criteria (fever, new epileptic seizures or neurological deficits, cerebral spinal fluid pleocytosis, specific alterations detected by neuroimaging or electroencephalography). The cause is unknown in approximately half of all cases. In the remainder, up to 50% are due to viral pathogens [1]. While specific antiviral treatment is available for a small subset of viral encephalitides—most notably acyclovir for herpes simplex encephalitis—therapy is merely supportive for most of them.

Patients at particular risk for viral encephalitis are those with congenital, acquired, or iatrogenic immunodeficiencies. Severe courses of viral encephalitides have—among others—been described after therapy with CD20-depleting agents [2, 3]. These agents act via direct depletion of pre-B and mature B-cells, therefore impairing the alloantibody response [4]. Furthermore, this effect may complicate the serological diagnosis, thereby delaying appropriate treatment [3].

In autoimmune encephalitis, the use of intravenous immunoglobulins (IVIG) is backed by controlled trials and has explicitly been recommended [5, 6]. They have also repeatedly been used as a prophylactic and therapeutic approach in viral encephalitides. Their use is mostly safe. Severe side effects are rare and include renal failure, thromboembolic events, and anaphylactic reactions. The latter are usually related to IgA deficiency [7]. However, their therapeutic efficiency in encephalitis is still a matter of debate. We conduct a systematic review on the safety and efficacy of IVIG in an adult and paediatric population with viral encephalitis.

## Methods

We conducted a systematic review and report it according to the Preferred Reporting Items for Systematic Reviews and Meta-Analyses (PRISMA) standards [8]. The main outcomes assessed were therapeutic efficacy (death/survival) and safety.

We performed a MEDLINE literature search using PubMed to identify all reports as of May 31, 2019 with no restrictions on start date using the search terms [“Encephalitis, Viral” (Mesh)] AND “Immunoglobulins, Intravenous” (Mesh) and [“Encephalitis, Viral” (Mesh)] and “Immunoglobulins, Intravenous/therapeutic use” (Mesh). Other databases searched include the Cochrane Database, Biosis Previews and the ClinicalTrials.gov website (search terms “viral encephalitis” AND “immunoglobulins”). Titles and abstracts of the reports obtained were screened for inclusion in the review using the following criteria: population with viral encephalitis (atypical viral infections such as JC-virus and slow-virus-infections were excluded); outcome and safety of IVIG therapy (reports on prophylactic IVIG use, intrathecal application of immunoglobulins, or use of IVIG-preparations that have been enriched for specific antiviral antibodies were excluded). Exclusion criteria were based on the intention to increase the homogeneity of the population under investigation.

Articles published in languages other than English, German, French or Spanish as well as duplicate studies, preclinical studies, editorials and reviews (except for secondary search) were excluded. Included were all case reports, case series, retrospective and prospective observational studies, and randomized controlled trials. A secondary search for other relevant articles was performed in the articles included after full-text analysis as well as in reviews on the topic.

The main outcomes assessed for observational studies, case series and clinical trials were efficacy and safety of the therapy. Efficacy was defined as survival. Safety was defined as number of severe adverse events. Secondary outcome parameters are listed in the results section if available from the reports. For case reports, the clinical outcome as stated in the respective paper is listed in Table 1.

**Table 1:** Case reports (max. 4 homogenous patients) included in the review

Author	Children (*n*)	Adults (*n*)	Immuno-suppressed (*n*)	Pathogen	Max. dose per day	Start of therapy (hospital day/day after symptom onset)	Duration of theray (days)	Mono- vs. add-on-therapy	Outcome
Akcakaya et al. [32]	1	0	0	EV	20 mg/kg	11/101	5	Mono	Good recovery
Barah [31]	1	0	0	hPV-B19	?	?/40	5	Add-on (steroids)	Full recovery
Borg et al. [44]	0	1	0	EBV	400 mg/kg	?/?	5	Add-on (aciclovir)	Full recovery
Caramello et al. [45]	0	1	0	JEV	400 mg/kg	?/?	5	Mono	Only slight deficits short term memory
Erol I [46]	1	0	0	hPV-B19	400 mg/kg	11/21	5	Add-on (steroids, acyclovir)	Clinical improvement (seizures)
Eyckmans et al. [47]	0	1	1	EV (Coxsackie-virus A16)	400 mg/kg	4/?	5	Add-on (aciclovir)	Marked improvement, intermitt confusion and language deficits
Fay AJ [48]	1	0	0	HHV-7	?	1/3	?	Add-on (aciclovir, steroids, plasma exchange)	Clinical improvement
Garzo-Caldas et al. [49]	0	1	1	EV	2 g/kg	?/?	1 (repeated bimonthly)	Add-on (methylprednisolone)	Dead
Geller et al. [50]	1	0	1	EV (Coxsackie-virus B4)	400 mg/kg	21/32	2	Add-on (aciclovir)	Full recovery
Georgescu et al. [51]	1	0	0	EV	?	?/?	10	Add-on (aciclovir, steroids)	Slow but favourable recovery
Golomb et al. [52]	0	1	0	EEEV	42 g	5/?	5	Add-on (aciclovir, steroids)	Full recovery
Greco et al. [53]	1	0	0	hPV-B19	400 mg/kg	?/3	5	Add-on (dexamethasone)	Severe ataxia
Hartmann et al. [54]	0	2	2	SLEV	400 mg/kg	7/8; 13/20	5	Add-on (INF alpha-2b)	1 × full recovery; 1 × residual dysarthria
Hindo et al. [55]	1	0	1	WNV	1 g/kg	4/11	3	Add-on (aciclovir, steroids;G-CSF); 1 × WNV-antibody-enriched IVIG	Dead
Hollander et al. [56]	0	1	1	WNV	?	7/21	1	Add (aciclovir)	dead
Kimura [57]	0	1	0	NV	4.2 g	3/5	3	Add-on (acyclovir, methylprednisolone)	Clinical improvement
Kleinschmidt-deMasters [58]	0	4	4	WNV	1 g/kg	?/?	1	Add-on (alpha-2b; ribavirin)	1 × no residual symptoms, 1 × mild residual symptoms, 2 × severe residual symptoms
Kleiter et al. [59]	0	1	0	TBEV	400 mg/kg	?/20	5	Mono	Clinical improvement with residual symptoms leading to early retirement
Lau et al. [60]	0	1	1	EBV	68 g	?/?	2	Add-on (ganciclovir)	Full recovery
Matsumoto et al. [61]	0	2	0	measles	5 g; 2,5 g	5/10; 5/11	3	1 × mono; 1 × add-on (aciclovir)	Full recovery
Miyagi et al. [62]	0	1	1	VZV	400 mg/kg	?/11	8	Add-on (aciclovir, ganciclovir)	Vegetative state
Morjaria et al. [3]	0	2	2	WNV	?	?/?; 11/?	?; 7	Add-on (aciclovir); mono	Dead
Nakano et al. [63]	1	0	0	JEV	5 g/d	?/?	3	Add-on (aciclovir, methylprednisolone)	Full recovery
Nolan et al. [64]	3	0	0	EV (EV71)	1 g/kg	5 to 9/?	2	Add-on (pleconaril, steroids)	?
Odessky et al. [65]	1	0	0	mumps	30 ml	1/5	5	Mono	Full recovery
Padate et al. [66]	0	1	1	EV	400 mg/kg	?/?	> 4	Add-on (aciclovir, ganciclovir)	Dead
Quartier et al. [67]	1	1	2	EV	?	?/?	?	Add-on (pleconaril)	Improvement with residual deficits
Rhee et al. [68]	0	1	1	WNV	400 mg/kg	4/?	2	Add-on (ganciclovir)	No neurological deficit
Saquib et al. [69]	0	1	1	WNV	1 g/kg	2/7	1,5	Add-on (aciclovir)	Full recovery
Schilthuizen et al. [70]	0	1	1	EV	40 g	?/?	1/week, ongoing	Mono	Full recovery
Shaheen et al. [71]	1	0	1	EV	500 mg/kg	?/?	1/week, ongoing	Add-on (aciclovir)	Significant neurological sequelae
Smudla et al. [72]	0	1	1	WNV	?	?/?	?	Add-on (aciclovir)	Complete recovery
Suga et al. [73]	1	0	0	mumps	1/kg	?/?	2	Add-on (methylprednisolone)	Bedridden
Ueno et al. [74]	0	1	1	CMV	5 g	?/?	3 (repeated monthly)	Add-on (aciclovir, ganciclovir)	Full recovery
Villace [75]	0	1	0	EBV	32 g/d	?/21	5	Add-on (acyclovir, dexamethasone)	Clinical improvement
Winston et al. [76]	0	4	4	WNV	500 mg/kg	?/6; ?/2; ?/8; 1/1	5;4;6;13	Add-on (aciclovir + INF-alpha 2b/INF-alpha2b (2x)/ribavirin)	Dead (2x)/full recovery (2x)
Xu [77]	0	1	0	CMV	400 mg/kg	28/?	5 (repeated 3-weekly)	Add-on (ganciclovir/valganciclovir, prednisone)	Clinical improvement
Yango et al. [78]	0	3	3	WNV	400 mg/kg	6/10; 1/3; 2/6	3; 4; 4	Add-on	Dead/discharge home/full recovery
Zaganas [79]	0	1	?	CMV	?	?/?	?	Add-on (ganciclovir, steroids)	Clinical improvement

*EV* enterovirus, *hPV-B19* human parvovirus B19, *EBV* Ebstein–Barr-virus, *JEV* Japanese encephalitis virus, *HHV-7* human herpesvirus-7, *EEEV* eastern equine encephalitis, *SLEV* St Louis encephalitis virus, *WNV* West Nile virus, *NV* norovirus, *TBEV* tick-borne encephalitis virus, *VZV* varicella zoster virus, *CMV* cytomegaly virus

Statistics were performed by JW using MedCalc^®^. Descriptive statistics were used. Where available, statistical results from group comparisons were extracted from the paper. If unavailable, odds ratios (OR) including 95% confidence intervals (CI) were calculated using individual patient data reported by the authors. Statistical significance was assessed using Fisher’s exact test.

A systematic assessment of the available evidence was conducted using the GRADE (Grading of Recommendations Assessment, Development and Evaluation) methodology [9, 10]. A meta-analysis was not performed due to the paucity of prospective randomised trials. A review protocol can be obtained from JW.

The study was exempt from ethical approval procedures by the Ethics Committee of Upper Austria.

## Results

**Fig. 1:**
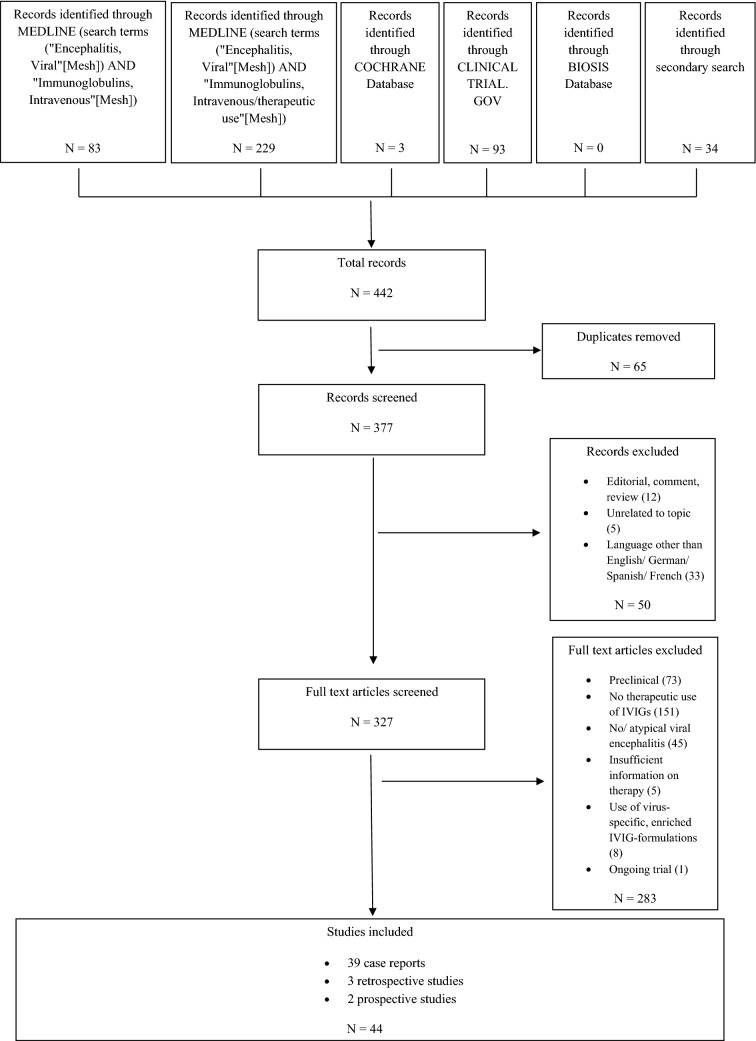
Flow-chart depicting the selection process of reports included in this review according to PRISMA guidelines

We screened a total of 377 studies, 44 of which were included (see Fig. 1): one prospective trial, one prospective case series, three retrospective observational studies, and 39 case reports. The case reports cover a total of 53 patients (37 adults, 16 children). 31 patients were immunosuppressed (27 adults): 17 post organ transplantation, 11 secondary to hematological malignancy, three due to autoimmune disease. The immunosuppressive drugs most frequently used included steroids (20 patients), calcineurin inhibitors (tacrolimus: 14 patients; ciclosporin: 5 patients), mycophenolic acid (18 patients), and anti-CD20 monoclonal antibodies (rituximab: nine patients; obinutuzumab: one patient). 21 patients had a combination of at least two of these agents.

The most common pathogens in adults (case reports) included West Nile virus (17 patients), enterovirus (four patients) and Epstein Barr virus (three patients), whereas, in children, enterovirus (eight patients), parvovirus B19 (three patients) and mumps virus (two patients) were most frequently reported. IVIG was used as monotherapy in seven patients and as add-on therapy in 46 patients in combination with acyclovir (21 patients), steroids (17 patients), interferon alpha-2b (nine patients), and ganciclovir (six patients). Other therapies applied included valganciclovir, plasma exchange, ribavirin, and pleconaril. In 13 patients, reduction of immunosuppression as part of the antiviral treatment was explicitly mentioned.

Modalities of IVIG-administration varied widely. The most frequently reported dose was 400 mg/kg (16 patients). The number of patients in whom this dose was used may have been higher as some authors only report total doses or do not specify the amount of IVIG applied. Application frequency varied between a single infusion and a continuous therapy in patients with ongoing immunosuppression.

IVIG-therapy was started between one and 101 days after symptom onset. For 28 patients, no information has been provided on the interval between symptom onset and the start of IVIG. For 20 patients, neither the interval between symptom onset nor hospital admission and the start of therapy was reported. Median treatment delay was 6.5 days (from hospitalization) and ten days (from symptom onset) in those patients that died, and 4.5 respectively eight days in those that recovered completely. This difference was even greater for West Nile Virus encephalitis patients with a median treatment delay of ten days from symptom onset in those who died and four days in those in whom symptoms remitted completely. No adverse effects of IVIG were explicitly mentioned.

Intensive care (ICU) dependency was explicitly reported for 26 patients. Most of these had a diagnosis of West Nile Virus encephalitis (13 versus 5 in the non-ICU group; *p* = 0.02) or enteroviral encephalitis (5 versus 8; *p* = 0.38). Unsurprisingly, more patients died in the ICU than in the non-ICU group (7 versus 2; *p* = 0.059). Of 53 patients, 44 were alive at the last follow-up. For 21 patients, full recovery was reported. In 20 patients, there were residual symptoms, in six of them severe ones (estimated modified Rankin scale 4–5). 9 patients had died (7 West Nile Virus encephalitis, 2 enterovirus encephalitis)—all of them had been immunosuppressed. For three patients, no follow-up information was available. For details on all case reports see Table 1.

Our search retrieved one prospective trial and one prospective case series. A prospective, randomized, placebo-controlled trial (RCT) investigated the use of IVIG harvested in a geographical region with high Japanese encephalitis (JE) prevalence (ImmunoRel^®^, 400 mg/kg/day for 5 days) in Nepalese children with suspected JE [11]. Saline solution was used as a placebo. However, only a small cohort consisting of 11 participants and 11 controls was tested as the study was designed as a pilot feasibility study. Endpoints consisted in survival, clinical outcome as Liverpool Outcome Score, Glasgow Coma Scale, and number of days of hospitalization at discharge, survival and clinical outcome at three to six months follow-up, and frequency of adverse events. Three serious adverse events are reported in JE patients: hypotension (one each in the IVIG and non-IVIG group) and melena (one in the IVIG-group). At discharge, one patient had died in the IVIG group, none in the control group (OR see Table 2). One patient in each group had recovered completely (OR 1, 95% CI 0–43.7). Equally, no significant differences concerning the other outcome parameter and safety endpoints at discharge or at follow-up were reported.

**Table 2:** GRADE assessment of the included trials, observational studies and case series

	No. of patients	Population	Dates	Pathogen	Design	Outcome	Death OR (95% CI)	Quality of evidence
Rayamajhi et al. [11]	22 (11 IVIG, 11 placebo)	Pediatric	05–09/2009	JE	Prospective trial	Death/survival; neurological outcome; adverse effects; duration of hospital stay; Glasgow Coma Score	At discharge: 3⋅29 (0⋅12–89⋅82) at 3–6 months: 0⋅45 (0⋅01–8⋅4)	Very low^a, b, j^
Zhang et al. [13]	80	Pediatric	03—09/2014	EV	Prospective case series	Clinical outcome; adverse effect	nc^k^	Very low^c^
Odessky et al. [15]	41 (27 IVIG, 14 controls)	Pediatric	1952	Measles	Observational study	Death/survival; neurological sequelae; adverse effects	0⋅75 (0.11–5⋅11)	Very low^b, c, g^
Neu [14]	72	Adults	1970-1979	Mixed	Observational study	Death/survival; neurological sequelae	0⋅032 (0⋅0033–0⋅3024)	Very low^b, d, e, f, g^
Wang et al. [80]	97 (Treatment/outcome data on 34 patients: 14 IVIG, 20 no IVIG)	Pediatric	04–12/1998	EV	Observational study	Survival/death	1⋅2 (0⋅26–5⋅59)	Very low^b, c, g, h, i, j^

Legend see Table 1. *nc *not calculable

^a^Unclear risk of detection bias

^b^Small sample size

^c^No control group and/or no randomized design

^d^Heterogeneous cohort

^e^High risk of selection or allocation bias

^f^High risk of detection bias

^g^Retrospective design

^h^Lack of information on part of cohort

^i^Lack of information in treatment protocol; possibly heterogenous treatment

^j^Loss to follow-up

^k^Study designed as a randomized controlled trial for the use of methylprednisolone in enteroviral encephalitis; no control intervention for IVIG—> study classified as case series

In addition to the prospective trial described in this review, a Cochrane Review on the use of IVIG in childhood encephalitis reports two more randomized trials in children with enteroviral and all-cause viral encephalitis [12]. We excluded these trials as the articles are in Chinese and not listed in any of the databases used for this review. Neither of them reports serious adverse events, nor do they provide data on the neurological outcome at discharge or follow-up. In a pooled analysis of these two trials, the Cochrane Review reports a significant advantage for some of the secondary endpoints (“length of hospital stay, time to resolution of fever, time to stop spasms, time to regain consciousness, time to resolution of neuropathic symptoms”) for those cohorts treated with IVIG. However, the quality of evidence was deemed to be very low.

A prospective case series focused on EV71-infection of the nervous system [13]. It was designed as a placebo-controlled randomized trial on the efficiency of a methylprednisolone pulse (10 mg/kg/d for three days; 40 patients in each of the verum and control group). Steroid treatment was not found to be beneficial in this trial. All patients received IVIG 1 g/kg/d for two days as part of the standard therapy. As all participants received IVIG, no conclusion on the efficacy of this treatment can be made. No IVIG-associated adverse effects were reported by the authors.

Furthermore, we found three retrospective observational studies. Wang et al. report on a cohort of 97 children (information on therapy obtained available in 34 patients) with EV71 encephalitis, 14 of whom were treated with IVIG: four of them died, ten survived. This compares to 15 survivors and five dead in the group who received supportive care only (OR 1.2; 95% CI 0.26–5.59). Dosage and timing of IVIG-therapy are not detailed – neither are secondary endpoints, including adverse events.

In a retrospective observational study the authors distinguish a group of 72 patients diagnosed as “viral meningitis, viral encephalitis and viral myelitis” [14]. The most frequent diagnoses included echovirus, (para)influenza, coxsackievirus, varicella virus, herpes simplex virus, and tick-borne encephalitis virus infections. However, the exact distribution of pathogens was not specified. In one-third of patients, the causative agent could not be identified. 58 patients were treated with IVIG (5 g/day for four successive days; treated at the author’s institution between 1975 and 1979) and had a significantly better outcome than the group who had not received IVIG therapy (treated at the same institution between 1970 and 1974). In the treatment group, one patient died and one was left with residual neurological deficits, compared to five patients who died and two who remained with residual deficits out of the 14 patients not treated with IVIG. The odds ratio for a lethal outcome was significantly lower in the group treated with IVIG (*p* = 0.0027), as was the odds ratio for incomplete neurological recovery (0.036, 95% CI 0.0062–0.207; *p* = 0.0002). A bias is most likely, not least because the two groups were treated during different time periods and it cannot be excluded from the manuscript that other therapeutic options had emerged in the interval. Furthermore, there is little information to determine the diagnostic accuracy. Hence, some cases may have been misdiagnosed as viral encephalitis.

In another retrospective non-randomized approach, the authors compare two groups of patients with measles encephalitis who received different IVIG-dosages [4 to 16 ml (12 patients) vs. 20 ml (15 patients); mg not specified] with a third group of 14 patients who did not receive IVIG [15]. The authors report that patients treated with IVIG reportedly had a better outcome, shorter hospital stays and lower mortality than the control group. However, the OR for death calculated from the numbers given in the report (two patients died in the control group, three patients in both treatment groups combined) does not reach significance; neither does the OR for incomplete neurological recovery (0.6, 95% CI 0.16–2.21). No adverse effects of IVIG treatment occurred.

Details on the trials, observational studies, and the case series including GRADE ratings are listed in Table 2.

## Discussion

Data on the efficacy of IVIG-therapy collected from case reports, case series, observational studies, and one RCT is heterogeneous. A clear superiority compared to supportive treatment could not be demonstrated. The data generated by the case series, observational studies and the RCT is of low-quality due to small and heterogeneous study populations and interventions, incomplete data, and possible selection, allocation, and detection bias. Most patients received IVIG as add-on therapy, thereby obscuring whether therapeutic effects were actually caused by this compound. Furthermore, the generalizability of the results to other pathogens and different socioeconomic settings is questionable. Hence, a general recommendation as to the use of IVIG in viral encephalitis cannot be made at this point.

The case reports reveal a strong association of fatal outcomes with pre-existing immunosuppression. Many patients included received combinations of steroids, calcineurin inhibitors, mycophenolate acid, and anti-CD20 therapeutics, leading to a combined deficiency of the T- and B-cell lines. In these patients, close monitoring of serum immunoglobulin levels to identify those who might benefit from replacement therapy may be advisable. In a cohort study of 8633 patients receiving rituximab, approximately half of patients whose immunoglobulin levels were investigated had hypogammaglobulinaemia [16]. The rate of severe infections in the study group was 28% in the 18 months following rituximab initiation and was highest in the group with hypogammaglobulinaemia. In those patients that received immunoglobulin replacement, a higher cumulative replacement dose was associated with a reduction of serious infections.

Side effects were reported in none of the case reports, case series, observational studies, or the RCT. This may be due to publication bias. In some patients, IVIG side effects may also have been mistaken for symptoms of the underlying disease.

Considering the high mortality and morbidity of encephalitis and the paucity of specific treatment options, more studies are urgently needed. One clinical trial investigating the role of early IVIG treatment in children with encephalitis (NCT02308982) is underway, investigating the effect of this therapy on clinical outcome (primary outcome measure). Future trials should analyze the following parameters as well:

### Are IVIG-preparations selected for their content of pathogen-specific antibodies more efficient than unselected preparations?

IVIG are plasma products of pooled IgG derived from multiple donors. They contain immunoglobulins directed at a wide variety of pathogens. Their exact composition depends on the prevalence of infectants in the geographic area of the population that contributed to the pool [17]. A twofold mode of action of IVIG has been proposed. First, IVIG may increase viral clearance due to antibody-dependent neutralization. Second, they have an immunomodulatory effect by mitigating hyperinflammation, which has been associated with a poor clinical outcome in viral encephalitis [18, 19]. This mechanism has been suggested to be independent of the presence of pathogen-specific antibodies [20]. Among the suggested mechanisms are impediment of CNS infiltration by pathogenic leukocytes, blockade of Fc receptors of macrophages, interference with complement activation, and modification of cytokine expression [21–23].

Experiments in mice demonstrated a dramatically reduced mortality in mice treated with IVIG-batches obtained from donors from a region endemic for West Nilve virus (WNV) compared to those obtained from US-donors harvested before WNV was introduced in the US [24]. Similar results have been described in a mouse model for tick-borne encephalitis (TBE) [25]. Several authors report successful application of these preparations in human patients.^65–67^ While their use seems intuitively convincing, data as to their efficiency is controversial: a recently published trial of 62 hospitalized WNV encephalitis patients randomized to receive Omr-IgG-am^®^ (an IVIG containing antibodies specific for WNV), standard IVIG, or normal saline showed no significant differences between groups receiving Omr-IgG-am compared with IVIG or saline for either the safety or efficacy endpoints [26]. Reasons may have included a small study population (the trial was terminated prematurely), delayed enrolment of participants (mean time from admission to infusion of study drug 2.7 days), and dosage (only a single infusion of the trial drug).

### Are alternative routes of immunoglobulin application more efficient than the intravenous administration?

Alternative routes of immunoglobulin application that have been described include intramuscular, subcutaneous, and intrathecal administration. Although meningeal inflammation enhances IVIG penetration of the blood–brain barrier, the amount of IVIG entering the CNS is unpredictable. Hence, direct installation of IVIG into the intrathecal space may be more efficient [17]. While some authors report cases successfully treated by these means, data from controlled trials are lacking [27–29]. Furthermore, (auto-)inflammatory reactions triggered by IVIG-binding to neuroglial epitopes might be a concern.

### What is the ideal dosing and timing of IVIG application?

Most articles included in this review report IVIG doses of 400 mg/kg. However, the approaches vary widely and are somewhat arbitrary as the ideal dosing has not yet been established. The mechanism of actions of IVIGs seems to be dose-dependent, with higher doses needed to obtain an immunomodulating effect, which may be desirable for some infections, but not for others [30]. In a murine WNV-model, the therapeutic effect of IVIG containing high anti-WNV antibodies correlated with its dose [24].

The same uncertainty applies to the timing of IVIG-therapy. We found a wide variation of the time span between symptom onset and therapeutic IVIG in the case reports included in this review. Delays to initiate therapy were most often associated with slowly progressive, unspecific clinical presentations that may occur with enteroviral or parvoviral infections, for example [31, 32]. Several reports suggest that neurotropic viruses are more susceptible to antibody-mediated clearance during the viraemic phase than to cell-mediated immunity once intracerebral spread has taken place. In a murine model, treatment with WNV-specific immunoglobulin was more efficient in the viraemic phase, before the neuroinvasive disease had occurred. IVIG has been postulated to efficiently prevent encephalitis if applied within the first four to six days after infection. Previous studies suggest that flavivirus may invade the brain as early as three days post-inoculation [17, 33–35]. Clearance of the virus during the viraemic phase may be the reason for the efficiency of prophylactic IVIG replacement as reported by Barmettler et al.[16]

However, some effect on mortality by inoculation of IVIG was seen even after the virus had reached the brain [24, 36]. Underlying antibody-mediated suppression of intracellular virus replication has been suggested [37, 38]. However, this effect may be pathogen-specific. The degree of postexposure prophylaxis for TBE correlated inversely to the time interval between infection and treatment with virus-specific immunoglobulins in mice. The authors conclude that protection against this disease is only possible before established CNS infection [39]. Data on a murine model of JE showed that the virus enters the brain from two to five days post-inoculation of JEV. This process takes place with the blood–brain barrier (BBB) intact. Disruption of the BBB induced by inflammatory cytokines and chemokines did not occur until day four [40]. Hence, peripheral application of IVIG may be efficient either before the virus enters the brain or after BBB disruption, but not in between. Analysis of the case reports included in this review showed a tendency towards better recovery when IVIG treatment was started earlier. However, controlled data backing this assertion is lacking.

### Does the efficiency of IVIG-therapy depend on the virus type?

The data obtained for this review do not permit conclusions as to which kind of viruses are more susceptible to IVIG therapy than others. However, the observations detailed above suggest that IVIG may eradicate those pathogens more efficiently that either remain bloodborne for an extended period and/or cause a significant BBB disruption. Many vector-borne viruses are transmitted via inoculation into the bloodstream where they may be neutralized by IVIG. As discussed above, they only remain bloodborne for a few days, necessitating a high level of suspicion and early commencement of therapy to obtain optimal results. On the other hand, studies on imaging characteristics in tick-borne encephalitis and West Nile virus encephalitis show that intraparenchymal contrast-enhancement as a sign of BBB disruption is uncommon in these diseases [41, 42]. Hence, these flavivirus species may be more difficult to treat with IVIG once they have entered the central nervous system.

Contrasting with the pathophysiology of vector-borne viruses, herpes simplex encephalitis is thought to occur via neuronal transmission. This may render IVIG therapy less efficient if administered early. However, diagnostic imaging frequently shows contrast-enhancement, probably rendering these patients amenable for add-on immunoglobulin treatment at this stage [43]. In this context, it would be interesting to obtain efficiency data on the combined use of acyclovir and IVIG in herpes simplex virus encephalitis.

In conclusion, only very low-quality evidence as to the clinical benefit and adverse effects of IVIG treatment in viral encephalitis exists. While IVIG application seems generally safe, its efficacy is still unclear. Hence, the indication and minutiae of IVIG-therapy in patients with viral encephalitis continue to rest on individual, case-specific decisions. RCTs in selected patient populations are needed to clarify its role in this severely affected cohort. Shortcomings of our review include the low-quality data of the reported studies due to small and heterogenous study populations and a high risk of bias, as well as incomplete data on individual patients and the confounding effect of multiple therapies.

## Declarations

### Availability of data and material

All data are included in the manuscript.
